# Risk Factors for Functional Outcomes of the Elderly with Intertrochanteric Fracture: A Retrospective Cohort Study

**DOI:** 10.1111/os.12512

**Published:** 2019-08-28

**Authors:** Jia‐bao Ju, Pei‐xun Zhang, Bao‐guo Jiang

**Affiliations:** ^1^ Department of Orthopaedics and Traumatology Peking University People's Hospital Beijing China

**Keywords:** Health‐related quality of life, Hip functional outcome, Intertrochanteric fracture

## Abstract

**Objective:**

To identify baseline factors relevant to functional outcomes and health‐related quality of life in the elderly with intertrochanteric fractures.

**Methods:**

For the present study, 168 patients with intertrochanteric fracture who were assigned to different treatments between January 2016 and December 2017 were retrospectively selected. Hip function was assessed by Harris hip score (HHS), and health‐related quality of life was evaluated by Barthel index (BI) of activities of daily living (ADL) and EuroQol 5‐dimensions (EQ‐5D) score, respectively. Data were analyzed by *t*‐test, ANOVA, Pearson's correlation, χ^2^‐test, and multivariate linear regression.

**Results:**

A total of 164 (97.6%) patients completed the follow‐up, with an average follow‐up time of 15.7 ± 6.9 months; 39 (23.8%) patients died during the follow‐up period and 125 (76.2%) patients were eligible for the functional analysis. HHS at final follow‐up of 125 patients was 71.8 ± 13.1, and the following were associated with hip functional recovery: age (−0.45, 95% confidence interval (*CI*) −0.73 to −0.18, *P* < 0.01), serum albumin (0.65, 95% *CI* 0.04 to 1.27, *P* < 0.05), and ADL at discharge (0.18, 95% *CI* 0.01 to 0.33, *P* < 0.05). The Barthel index at final follow up in this cohort was 80.2 ± 18.1, and multivariable linear regression analysis showed that age (−0.49, 95% *CI* −0.85 to −0.12; *P* < 0.05), ADL score at discharge (0.29, 95% *CI* 0.07 to 0.51; *P* < 0.05) and internal fixation (16.3, 95% *CI* 3.3 to 29.3; *P* < 0.05) were associated with ADL at final follow‐up. EQ‐5D at final follow‐up was 0.74 ± 0.2, with which HHS (0.012, 95% *CI* 0.011 to 0.013; *P* < 0.01) was positively associated.

**Conclusion:**

We identify several baseline factors associated with hip functional outcome, health utility, and ADL in the elderly after an intertrochanteric fracture, of which we could modify mutable factors to achieve better outcomes. These findings could help to inform treatment and functional prognosis.

## Introduction

Hip fracture is common and consists of one‐fifth of the operative work of an orthopaedic trauma center. It is a devastating health problem, associated with substantial mortality, morbidity, and costs. Millions of people are disabled as a result of hip fractures every year worldwide, which places a heavy burden on the healthcare system^1^, ^2^.

Fractures around the hip joint are associated with functional impairment and major disabilities. It is reported that over half of patients could not return to premorbid mobility status^3^. Factors influencing functional recovery have been a major concern of surgeons and physiotherapists. Previous studies have compared the efficacy and safety of hemi‐arthroplasty and intramedullary nails in treating unstable intertrochanteric fractures. The functional outcomes favored internal fixation from a long‐term perspective^4^, ^5^. In a randomized control trial comparing intramedullary nails and sliding hip screws for intertrochanteric fractures, intramedullary fixation was superior in improving activities of daily living (ADL) and health utility and in reconstruction to the pre‐fracture state^6^. A few studies have reported that patients with anemia at admission were not at increased risk of reduced ambulatory ability upon discharge from rehabilitation institutes^7^, ^8^, ^9^. In a perspective study, the results showed that older age and fragile economic status were associated with impaired mobility^10^. Notably, some studies have confirmed that pre‐injury function is positively correlated with functional recovery^11^, ^12^, ^13^, ^14^. Besides, cognitive impairment has been recognized as a negative prognostic factor for outcomes^11^, ^12^, ^15^, ^16^. In the analysis of bone turnover markers, unfavorable outcomes measured by Barthel index at discharge were associated with lower serum 25(OH)‐D levels^17^. In the radiological evaluation, the greater trochanter displacement following an intramedullary nail fixation is associated with a poor functional outcome^18^. Previous studies have identified some prognostic factors; however, these studies have exclusively focused on the relationship between recovery of ambulatory capacity upon discharge from hospital or rehabilitation institutes and baseline factors at admission with a short‐term follow up. Variables predicting the long‐term functional recovery and health utility of patients with hip fractures were still unclear.

Recently, the Fixation using Alternative Implants for the Treatment of Hip fractures (FAITH) investigators conducted an international, multicenter, allocation concealed, randomized controlled trial to assess the effects of sliding hip screws versus cancellous screws on reoperations over a 24‐month follow‐up^19^. Hip function and health‐related quality of life were secondary outcomes. The results demonstrated that smoking and displacement were risk factors for compromised hip function in patients with a sliding hip screw. Meanwhile, FAITH investigators used multilevel mixed models to identify potential factors associated with health‐related quality of life^20^. They found that female gender, higher ASA grade, and displaced fracture were inversely associated with health‐related quality of life. The outcomes showed that displaced fracture was associated with worse functional outcome. Higher age, female gender, higher BMI, higher ASA grade, displaced fracture, and closed reduction were associated with lower EQ‐5D score, indicating worse health utility.

Despite long‐term regular follow up over 24 months, the FAITH trial exclusively included patients with femoral neck fractures and excluded intertrochanteric fractures. Fractures around the trochanteric region were located outside of the hip joint capsule and were different from femoral neck fractures in relation to biomechanics and treatment options. Prognostic predictors for functional outcomes of patients with intertrochanteric fractures were still unknown. It was of great significance to identify additional factors associated with long‐term functional recovery, ADL, and health utility after an intertrochanteric fracture to optimize the care of these patients.

Therefore, the purpose of this retrospective study was to: (i) evaluate overall long‐term hip functional recovery and quality of life of patients after an intertrochanteric fracture; (ii) identify risk factors associated with hip functional recovery, ADL and health utility; and (iii) help to assess rehabilitation potential and modify mutable risk factors to reach satisfactory outcomes.

## Methods

### *Inclusion and Exclusion Criteria*

Inclusion criteria: (i): patients who were at 65 years of age and above with closed intertrochanteric fracture diagnosed by anterio–posterior and lateral plain film, CT or MRI; (ii): patients who had undergone intramedullary nail fixation (PFNA; AO, Switzerland); (iii): patients who had undergone hemi‐arthroplasty (Biomet, UK) or conservative therapy; (iv): the main outcome measures included Harris hip score (HHS), Barthel index of activity of daily living, European Quality‐five dimension; and (v): it is a retrospective case control study.

Patients were excluded if they met one of the following conditions: (i) patients with known metastatic cancer and pathology‐proven pathological fractures; (ii) fractures associated with poly‐trauma; (iii) younger than 65 years of age; (iv) patients were lost to follow‐up or with missing data.

The medical and nursing record of patients with intertrochanteric fractures were retrospectively retrieved from the Department of Medical Records between January 2016 and December 2017. The data included demographic information, perioperative images and laboratory tests, and treatments. Fractures were classified according to Arbeitsgemeinschaft fur Osteosynthesefragen/Association for the Study of Internal Fixation (AO/OTA) classification. Patients enrolled in the cohort were followed up with telephone interviews or clinical visits. The hip joint function was evaluated according to HHS, and quality of life was measured by EuroQol 5‐dimensions (EQ‐5D) and Barthel index (BI) of ADL.

### *Ethics Statement*

Written informed consent was obtained from all participants. All clinical investigations are conducted according to the principles expressed in the Declaration of Helsinki.

### *Surgical Techniques*

#### *Intramedullary Nail Fixation*

##### *Anesthesia*

Patients were given general or spinal anesthesia.

##### *Exposure*

Patients were placed in a supine position on a traction table. Satisfactory fracture reduction was achieved by limb traction with a slight abduction and pronation with the assistance of fluoroscopy. An incision was made 3 cm above the apex of the greater trochanter, and the gluteus medius was incised to expose it.

##### *Implantation*

The nail was implanted percutaneously. The guide needle was inserted into the medullary cavity under fluoroscopy. Then proximal part of the femoral shaft was reamed with the reamer, and the main nail was inserted into the medullary cavity. The guidewire was inserted into the femoral neck with the help of an aiming arm. An anti‐rotation spiral blade (PFNA; AO, Bettlach, Switzerland) was then inserted. The distal locking nail was achieved with the guidance of an aiming arm.

##### *Postoperation Treatment*

**Figure 1:**
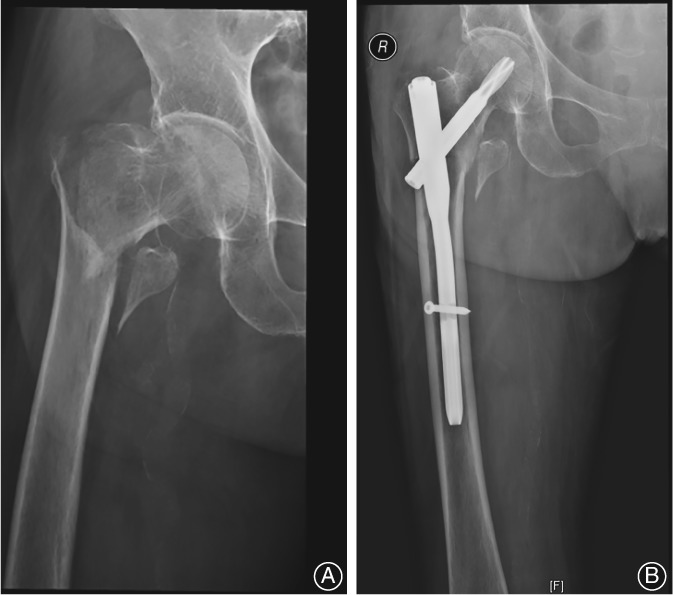
(A) Anteroposterior radiograph showed an unstable intertrochanteric fracture. (B) Radiograph made 1 week after fixation with proximal femoral nail anti‐rotation.

All patients were given prophylactic antibiotics (second generation of cephalosporins) within 24 h postoperation and low molecular weight heparin for thromboembolism prophylaxis. Patients were mobilized to bear full weight, as tolerated, for 6 weeks postoperatively (Fig. 1).

#### *Hemi‐arthroplasty*

##### *Anesthesia*

Patients were given general or spinal anesthesia.

##### *Exposure*

A lateral approach was used. Part of the gluteus medius and most of the anterior and lateral joint capsules were removed, then the femoral intertrochanteric fracture was exposed.

##### *Implantation*

The femur was cut obliquely 1.5 cm above the small trochanter. Then we took out the femoral head and exposed the medullary cavity of the femur, and tested the mold as the joint tightness was good. After injection of bone cement, the femoral prosthesis stem was inserted and an appropriate femoral head (Biomet, UK) was installed to maintain anatomical ante‐version and equal length of the contralateral limb. We placed additional wires around the intertrochanteric region to restore the fractured greater trochanter.

##### *Postoperation treatment*

**Figure 2:**
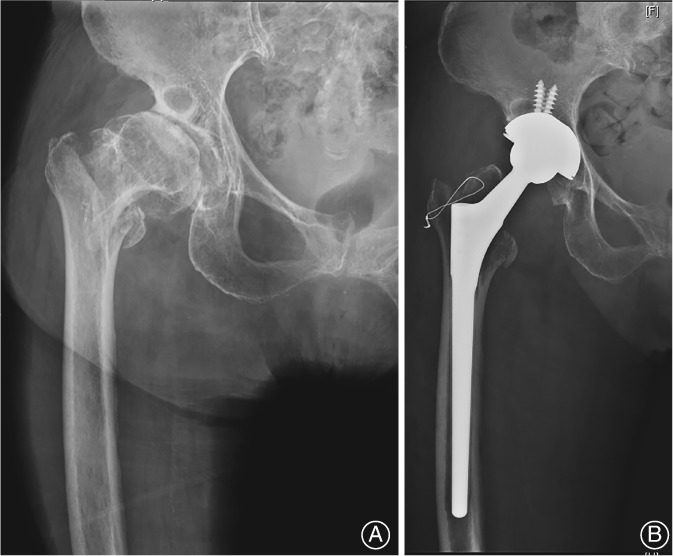
(A) Anteroposterior radiograph showed an unstable intertrochanteric fracture. (B) Radiograph made 1 week after a total hip replacement with additional wires to fix the fractured greater trochanter.

All patients received the same prophylactic antibiotics and thromboembolism prophylaxis as patients in the intramedullary nail group. Patients were mobilized to bear full weight, as tolerated, for 1 week postoperatively (Fig. 2).

#### *Conservative treatment*

**Figure 3:**
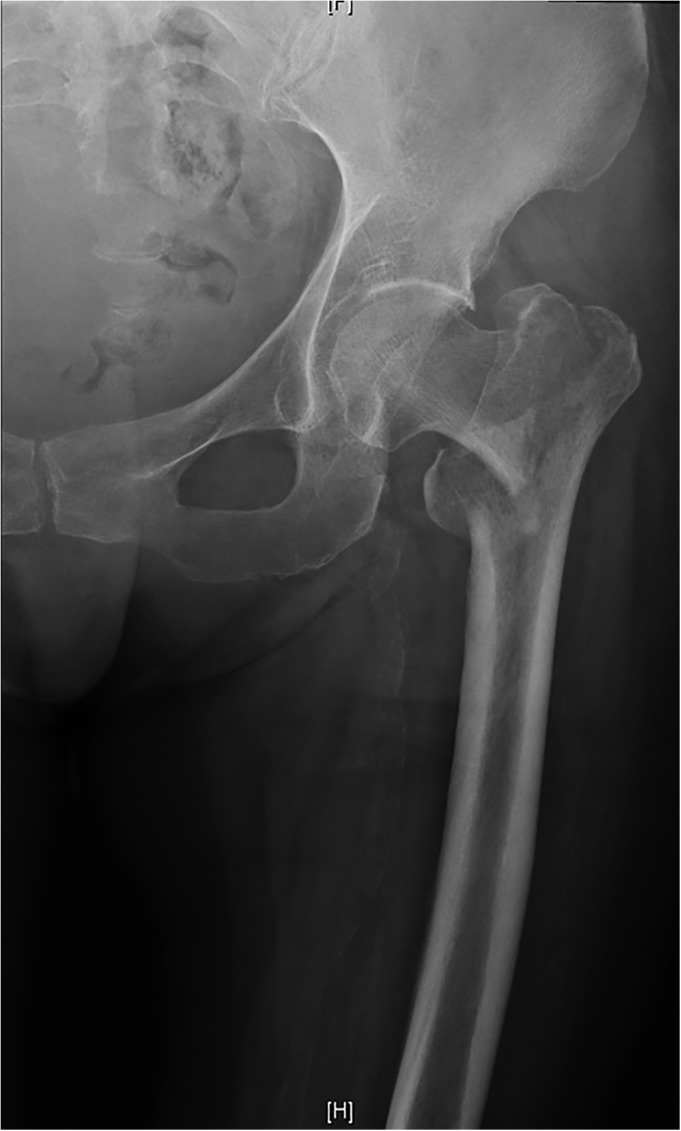
Anteroposterior radiograph showed an unstable intertrochanteric fracture.

Patients who refused surgical treatment and those with contraindications for surgery underwent conservative treatment. An anteroposterior radiograph showed an unstable intertrochanteric fracture of the left hip in a 43‐year‐old woman who fell at home 7 days previously (Fig. 3). She refused to undergo surgery and was immobilized for 2 months without further radiological assessment.

### *Outcome Measures*

#### *Harris Hip Score*

Harris hip score is a clinician‐administered tool and outcome scale developed in 1969 for patients with arthritis of the hip^21^. It contains 13 domains across four areas of pain, function, activity, and range of movement. Total scores range from 0 to 100, and higher scores indicate better hip function.

#### *EuroQol 5‐Dimensions*

The EuroQol 5‐dimensions (EQ‐5D) index comprises five dimensions: mobility, self‐care, usual activities, pain or discomfort, and anxiety or depression^22^. For each dimension, there are three levels: no problems, some/moderate problems, and extreme problems. The summary scores derived from EQ‐5D to the time trade‐off (TTO) represent a person's health utility, with lower scores indicating worse utility^23^.

#### *Barthel Index*

The Barthel index (BI) questionnaire contains 10 items: feeding, grooming, transfer, toilet use, mobility, dressing, going up and down stairs, bathing, bowel control, and bladder control. Scores for each activity reflect its relevance to the person's life. Items are summed with a total score range from 0 to 100. Higher scores reflect higher independence with ADL.

### *Statistical Analysis*

We used descriptive statistics to summarize all factors (frequencies and percentages for categorical variables, and means and standard deviation for continuous variables). The Pearson χ^2^‐test was used for comparison of categorical variables and the independent *t*‐test was used for continuous variables. Multivariate linear regression analysis with a 95% confidence interval was used to evaluate factors influencing hip function, ADL, and health utility. The significance level was set to 0.05 and all *P‐*values were two‐tailed. All analyses were performed using the SPSS software (Version 20.0, SPSS, IBM, Armonk, NY, USA).

## Results

### *General Results*

A total of 168 patients aged 65 years and above with intertrochanteric fractures were included in the cohort between January 2016 and December 2017; 112 (66.7%) were women and 56 (33.3%) were men, with patients aged from 65 to 93 years (80.1 ± 8.7). According to AO classification, 49 (29.2%) cases were classified as 31A1, 105 (62.5%) as 31A2 and 14 (8.3%) as 31A3. A total of 20 (11.9%) patients had recurrent contralateral hip fractures.

Of these 168 patients, 144 (85.7%) were treated with internal fixation, 10 (6.0%) underwent hip replacement, and 14 (8.3%) received conservative treatment. In the end, 164 (97.6%) patients completed follow‐up, with mean duration of 15.7 ± 6.9 months (Table 1). A total of 39 (23.8%) patients died during the follow‐up period. Finally, there were 125 (76.2%) patients who were eligible for analysis of risk factors relevant to functional outcomes and health‐related quality of life.

**Table 1:** Demographic information and clinical factors of patients with follow ups

	Age	Gender		Recurrent	Fracture type			ASA grade		BMI(kg/m^2^)	Hospital stay (d)	ADL‐D	Mortality
		Female	Male		A1	A2	A3	1–2	3–4				
IF	80.0 ± 8.5	95 (67.4%)	46 (32.6%)	17 (12.1%)	45 (31.9%)	84 (59.6%)	12 (8.5%)	88 (62.4%)	53 (37.6%)	23.2 ± 4.4	13.8 ± 6.7	35.3 ± 13.9	27(19.1%)
HR	81.9 ± 5.0	7 (77.8%)	2 (22.2%)	2 (22.2%)	1 (11.1%)	6 (66.7%)	2 (22.2%)	4 (44.4%)	6 (55.6%)	21.9 ± 5.1	15.3 ± 6.9	22.1 ± 7.4	2(22.2%)
NO	79.4 ± 12.8	0	5 (35.7%)	1 (7.1%)	1 (7.1%)	13 (92.9%)	0			21.9 ± 4.4	6.9 ± 3.0	26.1 ± 11.0	10(71.4%)
P	0.79	0.82		0.57	0.06			0.31		0.43	<0.01	<0.01	<0.01

ADL‐D, activity of daily living at discharge; ASA, American Society of Anesthesiologists; BMI, body mass index; HR, hip replacement; IF, internal fixation; NO, non‐operative.

### *Clinical Results*

Between groups of different treatments (intramedullary nail fixation, hip replacement, and conservative treatment), there were no significant differences in terms of mean age (*P* = 0.79), gender (*P* = 0.82), BMI (*P* = 0.43), fracture types (*P* = 0.06), ASA grade (*P* = 0.31), and recurrence of hip fracture (*P* = 0.57). Clinical outcomes in different surgical treatment groups are summarized in Table 2.

**Table 2:** Clinical outcomes in different treatment groups (mean±SD)

Groups	HHS	ADL‐F	EQ‐5D
IF	72.2 ± 13.2	81.6 ± 17.7	0.75 ± 0.18
HR	64.6 ± 10.4	65.7 ± 19.7	0.59 ± 0.24
NO	64.8 ± 10.0	66.3 ± 13.2	0.61 ± 0.11
*P*	0.19	0.02	0.03

ADL‐F, activity of daily living at final follow up; EQ‐5D, European quality of life‐5 dimensions; HHS, Harris Hip Score; HR, hip replacement; IF, internal fixation; NO, non‐operative.

### *Harris Hip Score*

Overall HHS at final follow up of 125 patients was 71.8 ± 13.1. Patients in internal fixation, hip replacement, and non‐operative treatment groups scored 72.2 ± 13.2, 64.6 ± 10.4, and 64.8 ± 10.0, respectively, without significant difference (*P* = 0.19). Patients in three different age groups (<70, 70–79, and ≥80 years of age) scored 80.0 ± 10.7, 72.4 ± 15.0, and 68.7 ± 11.2, respectively, and the difference was statistically significant (*P* < 0.01). Patients in the ASA 1/2 group scored 73.7 ± 13.9 and in the ASA 3/4 group scored 68.4 ± 11.2, with significant difference (*P* = 0.03). Patients with hypoalbuminemia (<35g/L) scored 73.7 ± 12.3, and with normal albumin scored 68.0 ± 13.7; the difference between groups was significant (*P* = 0.02). No statistically significant interrelations were observed between HHS and gender, as well as fracture type, treatment options, recurrence, Charlson comorbidity index (CCI), body mass index (BMI), and hemoglobin levels (Table 3).

**Table 3:** Association between patients’ baseline factors and Harris hip score

	Age			Gender		Fracture type			Treatment			Recurrent		ASA grade	
	<70	70–79	≥ 80	Female	Male	A1	A2	A3	IF	HR	NO	YES	NO	1–2	3–4
No.(%) of patients *n* = 125	17(13.6%)	45(36.0%)	63(50.4%)	84(67.2%)	41(32.8%)	42(33.6%)	72(57.6%)	11(8.8%)	114(91.2%)	7(5.6%)	4(3.2%)	14(11.2%)	111(88.8%)	77(61.6%)	44(35.2%)
Harris hip score	80.0 ± 10.7	72.4 ± 15.0	68.7 ± 11.2	70.9 ± 13.9	73.0 ± 11.5	73.1 ± 12.5	71.2 ± 13.9	68.0 ± 9.8	72.2 ± 13.2	64.6 ± 10.4	64.8 ± 10.1	70.2 ± 11.6	71.7 ± 13.3	73.7 ± 13.9	68.4 ± 11.2
*P*	<0.01			0.41		0.5			0.19			0.69		0.03	

	CCI				BMI				Alb		Hb		
	0	1	2	>2	<23	23–24.9	25–29.9	≥30	<35	>35	>120	90–120	<90
No.(%) of patients n = 125	54(43.2%)	47(37.6%)	19(15.2%)	5(4.0%)	57(45.6%)	31(24.8%)	25(20.0%)	12(9.6%)	78(62.4%)	47(37.6%)	49(39.2%)	62(49.6%)	14(11.2%)
Harris hip score	73.9 ± 11.4	69.3 ± 15.9	73.0 ± 8.7	62.6 ± 11.2	72.6 ± 11.5	71.6 ± 16.8	70.1 ± 13.6	69.8 ± 8.4	73.7 ± 12.3	68.0 ± 13.7	73.6 ± 11.9	70.7 ± 14.3	68.2 ± 11.2
P	0.13				0.83				0.02		0.32		

Alb, albumin; ASA, American Society of Anesthesiologists; BMI, body mass index; CCI, Charlson comorbidity index; Hb hemoglobin.

We used multivariable linear regression analysis to test factors that could serve as independent predictors for hip joint functional outcome at final follow up. The results showed that HHS was independently and inversely associated with age (*B* = −0.451; *P* < 0.01). Higher albumin levels (*B* = 0.654; *P* < 0.05) and ADL score at discharge (*B* = 0.175; *P* < 0.05) also emerged as independent factors predicting higher HHS at final follow‐up. None of the other variables that we tested, including hemoglobin levels, were predictive of HHS (Table 4).

**Table 4:** Linear regression analysis for variables predicting the Harris hip score

	Age	Alb	Hb	ASA	ADL‐D
*B* (95% *CI*)	−0.45 (−0.73 to −0.18)	0.65 (0.04 to 1.27)	−0.07 (−0.20 to 0.06)	−2.68 (−7.32 to 2.00)	0.18 (0.01 to 0.33)
*P*	0.002	0.036	0.28	0.26	0.034

ADL‐D, activity of daily living at discharge; Alb, albumin; ASA, American Society of Anesthesiologists; CI, confidence interval; Hb, hemoglobin.

### *Activities of Daily Living*

Overall Barthel index (BI) of ADL at final follow up of 125 patients was 80.2 ± 18.1. Patients in internal fixation, hip replacement, and non‐operative treatment groups scored 81.6 ± 17.7, 65.7 ± 19.7, and 66.3 ± 13.2, respectively, with significant difference (*P* = 0.02). Patients in three different age groups (<70, 70–79, ≥80 years of age) scored 90.0 ± 9.2, 82.2 ± 17.5, and 76.1 ± 19.2, respectively, and the difference was statistically significant (*P* = 0.01). Besides, there was a significant correlation between ADL at discharge and ADL at final follow up (*P* < 0.01). We observed no statistically significant interrelations between ADL at final follow up and gender, fracture type, recurrence, CCI, ASA grade, BMI, and albumin levels (Table 5).

**Table 5:** Association between patients’ baseline factors and ADL‐F

	Age			Gender		Fracture type			Treatment			Recurrent		ASA grade	
	<70	70–79	≥ 80	Female	Male	A1	A2	A3	IF	HR	NO	YES	NO	1–2	3–4
No.(%) of patients *n* = 125	17(13.6%)	45(36.0%)	63(50.4%)	84(67.2%)	41(32.8%)	42(33.6%)	72(57.6%)	11(8.8%)	114(91.2%)	7(5.6%)	4(3.2%)	14(11.2%)	111(88.8%)	77(61.6%)	44(35.2%)
ADL‐F	90.0 ± 9.2	82.2 ± 17.5	76.1 ± 19.2	79.1 ± 19.5	82.4 ± 14.8	80.5 ± 19.0	80.7 ± 18.2	75.9 ± 14.6	81.6 ± 17.7	65.7 ± 19.7	66.3 ± 13.2	80.5 ± 17.9	77.5 ± 19.8	83.1 ± 16.7	76.5 ± 19.7
*P*	0.011			0.34		0.71			0.02			0.56		0.05	

	CCI				BMI (kg/m^2^)				Alb		Hb		
	0	1	2	>2	<23	23–24.9	25–29.9	≥ 30	<35	>35	>120	90–120	<90
No.(%) of patients *n* = 125	54(43.2%)	47(37.6%)	19(15.2%)	5(4.0%)	57(45.6%)	31(24.8%)	25(20.0%)	12(9.6%)	78(62.4%)	47(37.6%)	49(39.2%)	62(49.6%)	14(11.2%)
ADL‐F	82.7 ± 15.9	77.5 ± 22.2	83.4 ± 9.0	67.0 ± 17.5	81.6 ± 15.5	80.3 ± 18.4	76.4 ± 24.0	81.3 ± 15.7	81.0 ± 18.0	78.9 ± 18.3	82.4 ± 17.2	78.6 ± 18.1	79.6 ± 21.4
*P*	0.15				0.69				0.55		0.56		

ADL‐F, activity of daily living at final follow up; Alb, albumin; ASA, American Society of Anesthesiologists; BMI, body mass index; CCI, Charlson comorbidity index; Hb, hemoglobin;

We performed a linear regression analysis to test factors that could serve as independent predictors for ADL at final follow up. The results showed that ADL at final follow up was independently and inversely associated with age (*B* = −0.49; *P* < 0.05). A higher ADL score at discharge (*B* = 0.29; *P* = 0.01) and internal fixation (*B* = 16.3; *P* = 0.01) also emerged as factors independently predicting higher ADL at final follow‐up. None of the other variables that we tested were predictive of ADL at final follow‐up (Table 6).

**Table 6:** Linear regression analysis for variables predicting the ADL‐F

	Age	ADL‐D	ASA	IF
*B* (95% *CI*)	−0.49 (−0.85 to −0.12)	0.29 (0.07 to 0.51)	−2.4 (−8.9 to 4.0)	16.3 (3.3 to 29.3)
*P*	<0.05	0.012	0.455	0.014

ADL‐D, activity of daily living at discharge; ADL‐F, activity of daily living at final follow up; ASA, American Society of Anesthesiologists; CI, confidence interval; IF, internal fixation.

### *EuroQol 5‐Dimensions*

EuroQol 5‐dimensions (EQ‐5D) in patients who survived at final follow up was 0.74 ± 0.2. Patients in internal fixation, hip replacement, and conservative treatment groups scored 0.75 ± 0.18, 0.59 ± 0.24, and 0.61 ± 0.11, respectively, with significant difference (*P* = 0.03). Patients in three different age groups (<70, 70–79, and ≥80 years of age) scored 0.83 ± 0.13, 0.77 ± 0.18, and 0.70 ± 0.19, respectively, and the difference was statistically significant (*P* = 0.02). Patients in the ASA 1/2 group scored 0.78 ± 0.16, and in the ASA 3/4 group scored 0.68 ± 0.21, with significant difference (*P* < 0.01). No statistically significant interrelations were observed between EQ‐5D at final follow up and gender, fracture type, recurrence, BMI, hemoglobin values, and albumin levels (Table 7).

**Table 7:** Association between patients’ baseline factors and EQ‐5D

	Age			Gender		Fracture type			Treatment			Recurrent		ASA grade	
	<70	70–79	≥ 80	Female	Male	A1	A2	A3	IF	HR	NO	YES	NO	1–2	3–4
No.(%) of patients *n* = 125	17(13.6%)	45(36.0%)	63(50.4%)	84(67.2%)	41(32.8%)	42(33.6%)	72(57.6%)	11(8.8%)	114(91.2%)	7(5.6%)	4(3.2%)	14(11.2%)	111(88.8%)	77(61.6%)	44(35.2%)
EQ‐5D	0.83 ± 0.13	0.77 ± 0.18	0.70 ± 0.19	0.73 ± 0.20	0.75 ± 0.15	0.75 ± 0.18	0.74 ± 0.20	0.70 ± 0.14	0.75 ± 0.18	0.59 ± 0.24	0.61 ± 0.11	0.69 ± 0.25	0.75 ± 0.17	0.78 ± 0.16	0.68 ± 0.21
*P*	0.016			0.56		0.77			0.028			0.27		0.006	

	BMI (kg/m^2^)				Alb		Hb		
	<23	23–24.9	25–29.9	≥ 30	<35	>35	>120	90–120	<90
No.(%) of patients *n* = 125	57(45.6%)	31(24.8%)	25(20.0%)	12(9.6%)	78(62.4%)	47(37.6%)	49(39.2%)	62(49.6%)	14(11.2%)
EQ‐5D	0.75 ± 0.16	0.74 ± 0.19	0.72 ± 0.24	0.75 ± 0.16	0.75 ± 0.19	0.72 ± 0.17	0.77 ± 0.16	0.72 ± 0.20	0.74 ± 0.18
*P*	0.95				0.3		0.41		

Alb, albumin; ASA, American Society of Anesthesiologists; BMI, body mass index; EQ‐5D, European quality of life‐5 dimensions; IF, internal fixation; HA, hemi‐arthroplasty; Hb, hemoglobin; NO, non‐operative treatment.

We performed a multivariable linear regression analysis to test factors that could serve as independent predictor for EQ‐5D at final follow up. This showed that EQ‐5D at final follow up was independently and positively associated with HHS (B = 0.012; *P* < 0.01). No other factors were found to be significantly associated with EQ‐5D at final follow up (Table 8).

**Table 8:** Linear regression analysis for variables predicting the EQ‐5D

	Age	HHS	HR	ASA
*B* (95% *CI*)	0.001 (−0.001 to 0.003)	0.012 (0.011 to 0.013)	−0.07(−0.14 to 0.004)	−0.03 (−0.07 to 0.005)
*P*	0.56	<0.01	0.07	0.09

ASA, American Society of Anesthesiologists; CI, confidence interval; EQ‐5D, European quality of life‐5 dimensions; HHS, Harris hip score; HR, hip replacement; IF, internal fixation.

### *Complications*

Within the follow‐up period, four patients developed pulmonary embolism, five patients were diagnosed with pulmonary infection, and seven patients had urinary tract infections. None had pressure sores.

## Discussion

Previous studies have indicated that the physical function of patients with intertrochanteric fractures was more impaired than for femoral neck fractures at discharge^24^. Prognostic predictors for hip function and health‐related quality of life in patients after an intertrochanteric fracture were major concerns of surgeons and physiotherapist. Our research evaluated the impact of baseline factors and treatments on functional outcomes and health utility in patients 65 years old and above with intertrochanteric fractures.

### *Clinical Outcomes*

The study showed that younger age, higher serum albumin, and ADL at discharge were significantly associated with better hip function at final follow up, measured by HHS. In addition, we disclosed that younger age, higher ADL at discharge, and internal fixation (compared with conservative treatment) were related to higher ADL at final follow up as measured by the Barthel index. Finally, we revealed that post‐fracture EQ‐5D scores indicated that overall health utility was positively associated with hip joint function.

### *Harris Hip Score*

In our study, hemoglobin levels did not emerge as an independent risk factor for impaired hip function both in simple and multivariable regression analysis. A recent meta‐analysis reviewed studies on variables associated with hip joint function and concluded that anemia on admission was associated with poor functional outcomes with a weak level of evidence. It suggested that patients with low hemoglobin on admission were more likely to be frail and had less muscle strength than those without anemia^25^. However, a recent randomized controlled trial found that a more liberal blood transfusion policy did not result in a better recovery of ADL^26^. In fact, hemoglobin levels after acute fracture did not accurately reflect frailty. Fractures around the hip joint resulted in substantial amount of blood loss, and hemoglobin level stabilized shortly due to concentration and then declined over time^27^. Hemoglobin level fluctuated dramatically due to physiological stress as a result of fractures and iatrogenic resuscitation after acute fractures. Therefore, hemoglobin level was not a reliable predictor of frailty. Besides, hemoglobin level tested at different post‐injury time‐points could contribute to the heterogeneity across patients and studies.

In a study including 499 elderly patients with hip fractures, Mizrahi *et al*. found that albumin levels could not independently predict better functional outcomes^28^. However, a larger retrospective study of 17 651 patients reported that hypoalbuminemia was associated with increased mortality and complications compared with patients with normal albumin levels^29^, ^30^. Complications limited the rehabilitation of hip joints, resulting in a compromised functional outcome. This discrepancy may be explained by the small sample size and short follow up. Our study found that albumin was an independent prognostic factor for hip function. Specifically, albumin reflected nutritional status in the past few months, and remained stable within a short time after acute fracture. To the best of our knowledge, albumin was a reliable indicator for frailty, and could serve as a prognostic factor for post‐fracture hip joint function.

Garden classification of femoral neck fractures was based on the degree of displacement, which was judged by AP radiograph by determining the relationship of the trabecular in the femoral head to those in the acetabulum. Garden III and IV type indicated more displacement, and in that case, vessels that supplied to the femoral head along the neck would be damaged. The results of the FAITH trial strongly supported that displacement was associated with poor hip function. Femoral head necrosis was proposed as the underlying mechanism for the association between Garden classification and functional outcome^31^. Previous studies indicated that compared with femoral neck fractures, intertrochanteric fractures were an independent risk factor for poorer hip function^32^. Classification of intertrochanteric fractures as type A1, A2, and A3 was done to imply the stability of fractures, to give guidance for different surgical treatments, and to predict the long‐term clinical outcome of implants. Accordingly, among the prerequisites for stable fixation, type of intertrochanteric fracture had little implication for functional recovery. With regard to relatively small sample sizes, further research should be conducted to determine the correlation of fracture type with functional outcomes.

In a study of 303 patients with intertrochanteric fractures, Tang *et al*. found that HHS decreased in the elderly patients due to aging. Patients below 70 years of age had a mean HHS of 86.7 years, patients between 85 and 90 years old scored 79.3, and those older than 90 years only scored 77.1 on average^33^. The results were consistent with our findings. Older age was an independent and non‐modifiable risk factor for hip function.

## Activities of Daily Living

We found that activities of daily living measured by Barthel index (BI) at final follow up was inversely related to older age. Hip joint function declined with aging, and compromised function limited mobility, which resulted in impaired ability to perform daily tasks. ADL at final follow up was associated with ADL at hospital discharge. Ishidou (2017) found that an increased risk of BI deterioration was associated with worse BI at discharge, which suggested that early rehabilitation could improve functional prognosis^34^. Approximately 30% of patients underwent rehabilitation exercises in specialized institutions after surgery. Better ADL at discharge suggested better hip function and mobility, which allowed patients to ambulate earlier and enhanced the muscular strength of the lower limbs.

## EuroQol 5‐Dimensions

Our study suggested that health utility, measured by EQ‐5D, was strongly associated with hip function. A systematic review of studies investigating health status or health‐related quality of life following hip fractures found that mental status, pre‐fracture function, comorbidity, female gender, nutritional status, postoperative pain, length of hospital stay, and complications were associated with health‐related quality of life with strong evidence^35^. In our study, we identified no other risk factors of EQ‐5D for elderly patients after an intertrochanteric fracture, which indicated that hip function was a close predictor for health‐related quality of life. Well‐recovered hip function enjoyed better mobility and made it easier to fulfill daily activities, such as having meals and taking showers. Health‐related quality of life was substantially associated with hip function. Due to retrospective analysis, data regarding the pre‐fracture function and mental status were lacking, and we cannot confirm their predictive value.

### *Strengths and Limitations*

This study had several strengths. First, to the best of our knowledge, it is the first to focus on the baseline factors associated with hip function, ADL, and health‐related quality of life in a retrospective cohort of patients after an intertrochanteric fracture. In addition, inclusion of three different outcome measures contributed to the validity of the results. The study was strengthened by the more than 12‐month follow‐up period, which provided a long‐term assessment of the patients.

Despite these strengths, the study was limited in its retrospective nature. Patients were not assigned to the groups randomly, which could have biased the results to some extent. It is possible that for larger groups of patients with various treatments, HHS differences would be significant and have sufficient statistical power. Finally, BI of ADL scales was the most widely used index in the previous literature, which has been validated for use in elderly patients. Nevertheless, it was not specifically used in the hip fracture population and could be affected by comorbidities. In comparison to generic scales, disease‐specific scales, including HHS and WOMAC, were more specialized in disease state. However, disease‐specific measures used in musculoskeletal research have focused on patients with osteoarthritis, and not specifically on hip fractures. In fact, functional outcomes also depended on the death rate as patients may have recovered if they remained alive.

### *Conclusion*

In this retrospective analysis, to begin with, we reviewed the functional outcomes and health‐related quality of life of patients after rehabilitation following an intertrochanteric fracture. Second, we identified age, albumin, and ADL at discharge as relevant factors for functional recovery. Age, ADL at discharge, and fixation type emerged as predictors for ADL at final follow up. In the end, we found that health utility was strongly associated with hip function.
